# An Asian Patient with Myoclonus-Dystonia (DYT11) Responsive to Deep Brain Stimulation of the Globus Pallidus Internus

**DOI:** 10.1155/2014/937095

**Published:** 2014-02-09

**Authors:** Akinori Uruha, Katsuo Kimura, Ryoichi Okiyama

**Affiliations:** ^1^Department of Neurology, Tokyo Metropolitan Neurological Hospital, 2-6-1 Musashidai, Fuchu, Tokyo 183-0042, Japan; ^2^Department of Clinical Development, Translational Medical Center, National Center of Neurology and Psychiatry, 4-1-1 Ogawa-Higashi, Kodaira, Tokyo 187-8551, Japan; ^3^Department of Clinical Neurology and Stroke Medicine, Yokohama City University Graduate School of Medicine, 3-9 Fukuura, Kanazawa-ku, Yokohama, Kanagawa 236-0004, Japan

## Abstract

We describe the case of a 42-year-old Japanese woman with childhood-onset myoclonus, dystonia, and psychiatric symptoms, including anxiety, phobia, and exaggerated startle response. The diagnosis was confirmed as myoclonus-dystonia (DYT11) by identifying a mutation in the gene encoding **ε**-sarcoglycan. Interestingly, while motor-related symptoms in DYT11 generally improve with alcohol ingestion, the patient's symptoms were exacerbated by alcohol intake. Her severe and medically intractable symptoms were alleviated by bilateral deep brain stimulation of the globus pallidus internus, with myoclonus and dystonia scores showing 70% improvement after the surgery compared to presurgical scores. This is the first report of a genetically confirmed case of DYT11 in Japan. This paper together with other recent reports collectively demonstrates that DYT11 patients are distributed worldwide, including Asia. Thus, a diagnosis of DYT11 should be considered when clinicians encounter a patient with childhood-onset myoclonus and/or dystonia with psychiatric symptoms, regardless of ethnic background.

## 1. Introduction

Myoclonus-dystonia is an inherited autosomal dominant movement disorder. Although genetically heterogeneous, most hereditary forms of myoclonus-dystonia are due to mutations in the *ε*-sarcoglycan gene (*SGCE*) [1]. Myoclonus-dystonia caused by the *SGCE* mutation is classified as DYT11 (OMIM 159900) and characterized by childhood-onset myoclonus, dystonia, and psychiatric symptoms. The psychiatric symptoms are variable, including anxiety, obsessive-compulsive behaviors, and depression. While many patients with DYT11 have been described in Caucasian population, only a few cases have been reported from other populations [2–6]. Here, we describe the first Japanese patient with genetically confirmed DYT11. Although the patient had severe manifestations of motor symptoms, bilateral deep brain stimulation of the globus pallidus internus (GPi-DBS) has successfully alleviated them.

## 2. Case Report

A 42-year-old Japanese woman was admitted to our hospital with complaints of involuntary jerking movements of the trunk and hands. Many of her symptoms appeared in early childhood; these include anxiety, fear, and jerking movements of her hands, which her parents noticed when she was 5 years old. Since then, she has also been easily and intensely startled by abrupt touches and loud sounds. These symptoms have been accompanied by mild torticollis and scoliosis since she was 7 years old, with axial jerks appearing in her teenage years. Furthermore, she developed an extreme fear of showing the involuntary movements to others, thus limiting her social interactions. Motor symptoms developed gradually, but, by the age of 40, she was severely disabled and unable to properly perform daily living activities such as eating, drinking, and writing. In the past, several clinicians had tried prescribing psychotropic drugs to treat what they believed was a psychiatric disorder rather than a movement disorder, hoping that by alleviating the psychiatric symptoms the motor symptoms might also improve. However, these drugs had no effect on any of her motor symptoms.

Upon entering our hospital, neurological examination showed myoclonic movements in axial and shoulder girdle muscles. Additionally, moderate truncal and mild cervical dystonia were seen. Both the myoclonus and dystonia were aggravated by psychological stress. The patient's scores on the Unified Myoclonus Rating Scale (UMRS) were 29 (rest), 55 (action), 15 (stimulus), and 13 (functional test), while her score on the Burke-Fahn-Marsden Dystonia Rating Scale (BFMDRS) was 14. Alcohol ingestion did not improve, but rather exacerbated, her motor symptoms. Exaggerated startle response was repeatedly observed in response to sudden acoustic and tactile stimuli. Surface electromyographic recordings of the myoclonus showed synchronous activities predominantly in axial and shoulder girdle muscles. There was no electrophysiological evidence of cortical myoclonus. Routine brain magnetic resonance imaging findings showed that the brain was structurally normal. Psychiatric examination demonstrated that she had phobic anxiety disorder with panic attacks.

**Figure 1:**
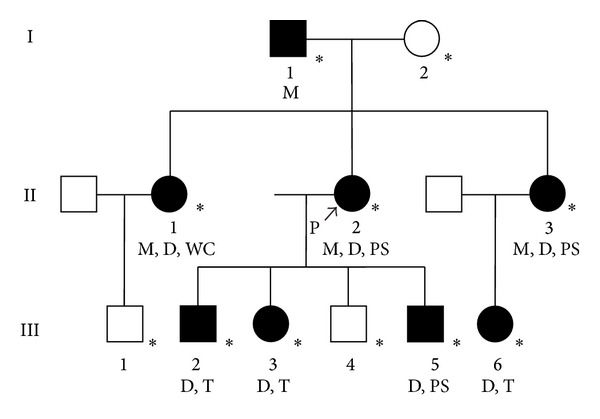
Pedigree structure of the family. I-1 (74 years) had mild myoclonus in his neck induced by psychiatric stress. II-1 (45 years) had cervical dystonia, writer's cramp, and mild myoclonus in her upper limb, neck, and voice. Alcohol intake does not improve her symptoms, according to interview. II-2 (42 years) is the proband of this family and has an *ε*-sarcoglycan gene mutation causing DYT11. Details are described in the text. II-3 (40 years) had cervical dystonia, mild action myoclonus in her right hand, and psychiatric symptoms, which were diagnosed as schizophrenia by psychiatrists. She often feels fear. In III-2 (21 years) and III-3 (13 years), cervical dystonia and minimal postural tremor of hands were observed. III-5 (7 years) had cervical and lingual dystonia and water phobia. III-6 (13 years) showed cervical dystonia and mild postural tremor in her upper limbs. The symptoms of the first and third generations were milder than those of the second generation. I-2 (72 years), III-1 (4 years), and III-4 (12 years) do not have any motor or psychiatric symptoms. *∗*: directly examined individual; P: proband; M: myoclonus; D: dystonia; PS: psychiatric symptom; T: postural tremor; WC: writer's cramp.

Direct interviews and physical examinations of the patient's family members revealed seven affected individuals (Figure 1). The manifestations of symptoms were variable among the family members. Motor symptoms included myoclonus, dystonia, writer's cramp, and postural tremor. The younger sister (II-3) and the youngest son (III-5) of the proband had psychiatric symptoms: II-3 was diagnosed with schizophrenia by psychiatrists, and III-5 had water phobia. The proband showed the most disabling symptoms; however, the symptoms of her siblings were still relatively severe compared to other generations.

After obtaining informed consent, all exons and exon-intron junctions of *SGCE* were analyzed by direct bidirectional sequencing of DNA extracted from her peripheral blood lymphocytes. A heterozygous mutation c.233-1G > A in intron 2 was detected. This mutation was described previously in French families as a pathogenic splice site mutation [7, 8].

As motor symptoms of the proband were severe and medically intractable, bilateral GPi-DBS was performed. The bilateral GPi-DBS improved her motor symptoms remarkably, with both UMRS and BFMDRS scores being about 70% lower 2 months after the operation compared to before the operation. Her postoperative UMRS scores were 9 (rest), 16 (action), 5 (stimulus), and 3 (functional test) on UMRS, while her postsurgical BFMDRS score was 4. Her psychiatric symptoms also seemed to be alleviated postoperatively, although no formal objective assessment was performed. The efficacy of GPi-DBS has been maintained over the 3-year follow-up period.

## 3. Discussion

The present patient was diagnosed with DYT11, as confirmed by the reported *SGCE* mutation. Some of the family members also had motor and psychiatric symptoms consistent with DYT11 [6]. Although genetic analysis was not performed on the family members, it is clinically suggested that they are also DYT11 patients. The manifestations of the symptoms in affected family members were variable, and symptoms of the first and third generations were relatively mild compared to those of the second generation. This intrafamilial phenotypic heterogeneity presumably reflects involvement of maternal imprinting, which is known to occur in the human SGCE gene [9].

Alcohol intake improves motor symptoms in most patients with DYT11 [6]. Unexpectedly, the patient in this case showed exacerbated motor symptoms in response to alcohol ingestion. While there is another report of a DYT11 patient showing “alcohol-induced dystonia,” the cause is unclear [10]. It should be noted that the patient in our report cannot inherently drink much alcohol and became inebriated on just the small amount of alcohol administered at the ingestion test. There is a possibility that heavy drinking may rather exacerbate motor symptoms in DYT11, although this is a speculation based only on a single case.

Bilateral GPi-DBS is considered an effective treatment for DYT11, with patients showing >50–80% improvement in both dystonia and myoclonus scores [6, 11]. Although DBS of the ventral intermediate thalamic nucleus (Vim) has also been reported to be effective, there are less stimulation-induced adverse events with GPi-DBS compared to Vim-DBS [12]. Thus, GPi-DBS seems to be a preferable therapeutic option. Additionally, the successful result of GPi-DBS in the present patient is encouraging for the use of GPi-DBS as a treatment option for patients with severe DYT11. The exact mechanisms underlying the success of GPi-DBS for DYT11 are not well understood, even though there is some anecdotal electrophysiological evidence showing the synchronization of pallidal local field potentials and electromyographic activities during spontaneous dystonic and myoclonic movements [13, 14].

To our knowledge, this is the first genetically confirmed case of DYT11 in Japan. This case together with other recent reports collectively demonstrates that DYT11 patients are distributed worldwide, including Asia [2–6]. Thus, DYT11 should be considered as a differential diagnosis when clinicians encounter a patient with childhood-onset myoclonus and/or dystonia with psychiatric symptoms, regardless of ethnic background.
